# Comparative analysis indicates regulatory neofunctionalization of yeast duplicates

**DOI:** 10.1186/gb-2007-8-4-r50

**Published:** 2007-04-05

**Authors:** Itay Tirosh, Naama Barkai

**Affiliations:** 1 Department of Molecular Genetics, Weizmann Institute of Science, 76100 Rehovot, Israel; 2 Department of Physics of Complex Systems, Weizmann Institute of Science, 76100 Rehovot, Israel

## Abstract

**Background:**

Gene duplication provides raw material for the generation of new functions, but most duplicates are rapidly lost due to the initial redundancy in gene function. How gene function diversifies following duplication is largely unclear. Previous studies analyzed the diversification of duplicates by characterizing their coding sequence divergence. However, functional divergence can also be attributed to changes in regulatory properties, such as protein localization or expression, which require only minor changes in gene sequence.

**Results:**

We developed a novel method to compare expression profiles from different organisms and applied it to analyze the expression divergence of yeast duplicated genes. The expression profiles of *Saccharomyces cerevisiae *duplicate pairs were compared with those of their pre-duplication orthologs in *Candida albicans*. Duplicate pairs were classified into two classes, corresponding to symmetric versus asymmetric rates of expression divergence. The latter class includes 43 duplicate pairs in which only one copy has a significant expression similarity to the *C. albicans *ortholog. These may present cases of regulatory neofunctionalization, as supported also by their dispensability and variability.

**Conclusion:**

Duplicated genes may diversify through regulatory neofunctionalization. Notably, the asymmetry of gene sequence evolution and the asymmetry of gene expression evolution are only weakly correlated, underscoring the importance of expression analysis to elucidate the evolution of novel functions.

## Abstract 2

Comparison of the expression profiles of *S. cerevisiae* duplicate pairs with that of their pre-duplication orthologs in *C. albicans* identified a class of genes that may present cases of regulatory neofunctionalization.

## Background

Current genomes were shaped by numerous duplications of single genes, chromosomal segments and even entire genomes [1-3]. In most cases, one copy of the duplicated gene is rapidly lost either by deletion or through mutations ('nonfunctionalization'), reflecting the lack of selection for each individual copy. In other cases, however, both duplicates may survive despite the initial redundancy and become fixed in the genome. The retention of both duplicates over millions of years implies that they confer an advantage such that deletion of either copy will cause a reduction in fitness.

While the evolutionary advantage of duplicates retention is usually difficult to ascertain, several models have been suggested [4]. First, duplicates could be retained due to selection for robustness through redundancy [5], although this view has been frequently challenged [6,7]. Second, selection for high protein dosage may favor the presence of two gene copies [8]. In these cases, similarity between the two copies can be maintained by negative selection or by gene conversion. Third, each of the duplicates may specialize in a subset of the ancestral functions, such that the ancestral functions require the activity of both genes ('subfunctionalization'). Fourth, one of the duplicates may retain the ancestral functions while the other evolves to perform a novel function ('neofunctionalization'). Identifying these scenarios and, in particular, recognizing cases of neofunctionalization may provide new insights into genome evolution, since duplications are believed to constitute the main origin of novel functions.

The term neofunctionalization refers to the acquisition of a novel function. However, it is typically difficult to define what the function of a gene is, and what constitutes a novel function. One obvious aspect of gene function is the catalytic activity performed by encoded enzymes. A broader definition of gene function, however, should include other aspects, such as protein localization, interactions with other proteins and expression patterns. These features are usually difficult to infer from the protein sequence, but the abundance of functional genomics datasets and the advent of microarray technology can now be used to analyze these properties directly. Of particular interest are the expression patterns of genes in various conditions. Changes in expression patterns have been suggested to be the primary source of phenotypic divergence among related species [9]. Such regulatory changes can have a profound effect on the function of a duplicated gene and, thus, lead to the preservation of a duplicate pair [10-12]. We refer to this scenario, where one copy of a duplicate pair diverges in expression pattern thereby facilitating the acquisition of a novel function, as regulatory neofunctionalization.

The yeast *Saccharomyces cerevisiae *is an excellent model to study the diversification of duplicate gene pairs. First, extensive functional annotations and expression data are available for *S. cerevisiae*. Second, the *S. cerevisiae *ancestor has undergone a whole genome duplication (WGD) event about 100 million years ago [13]. Sequencing of the pre-duplication yeast, *Kluyveromyces waltii*, identified hundreds of duplicate gene-pairs that were retained following this WGD event [2]. Many of these duplicate pairs accumulated extensive divergence and evolved new or altered functions. For example, sequence comparisons between *S. cerevisiae *duplicate pairs and their single orthologs from *K. waltii *revealed that in a significant portion of the duplicate pairs (115 out of 457), one copy has diverged in sequence significantly faster than the other copy [2]. This was taken as evidence for neofunctionalization, with the more conserved copy retaining the ancestral function and the other copy evolving to perform a new or altered function. A similar analysis of expression patterns may reveal additional cases of regulatory neofunctionalization.

Recent studies reported that 40% of the duplicate pairs in *S. cerevisiae *differ significantly in their expression patterns [14,15]. However, to identify cases of neofunctionalization, the expression pattern of each of the copies must be compared with the ancestral expression pattern. To circumvent this problem, Gu *et al*. [15] focused on gene families that contain a duplicate pair and at least one additional gene that was assumed to represent the ancestral expression pattern. In the absence of data about the expression of the ancestral genes, however, the validity of this assumption is difficult to assess.

Here we analyze the diversification of yeast duplicates by directly comparing their expression patterns in a post-duplication species (*S. cerevisiae*) to those of their single orthologs in a pre-duplication species (*Candida albicans*) as a proxy for the ancestral gene expression. We first describe a general method for comparative analysis of expression profiles from related organisms. We apply this method to compare large datasets from hundreds of microarray experiments in both yeast species. Focusing on duplicate gene pairs, we identify 43 duplicated gene pairs with asymmetric rates of expression divergence. These gene pairs are likely to present instances of regulatory neofunctionalization. Notably, the level of sequence divergence in many of these duplicates is similar, emphasizing the need to include gene regulation as a complementary means for analyzing functional divergence.

## Results

We first describe our method for comparison of expression profiles between one-to-one orthologs from two organisms, and later extend it to examine the expression conservation of duplicate gene pairs.

### A novel method for comparative analysis of gene expression

**Figure 1:**
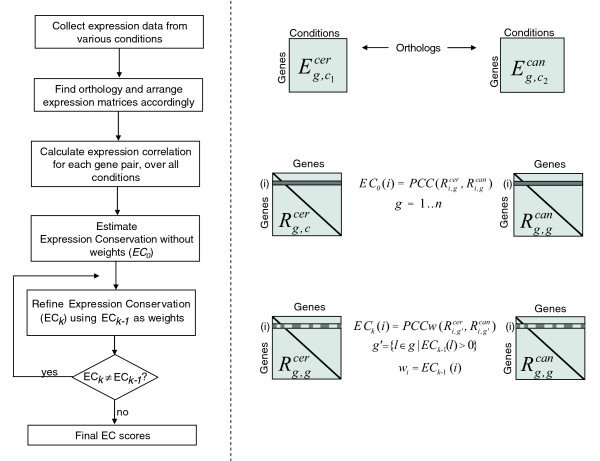
Method for comparative analysis of gene expression. Given expression matrices for two species where rows correspond to genes and columns correspond to conditions, we first find one-to-one ortholog matches between the two species and arrange the matrices such that equivalent rows represent the expression patterns of orthologs. Note that after this step the two matrices have the same number of rows, but not necessarily the same number of conditions and the conditions are not comparable. Next, the Pearson correlation coefficient (PCC) is calculated for each pair of genes over all conditions, generating the correlation matrices Rg,gcer
 MathType@MTEF@5@5@+=feaafiart1ev1aaatCvAUfeBSjuyZL2yd9gzLbvyNv2Caerbhv2BYDwAHbqedmvETj2BSbqee0evGueE0jxyaibaiKI8=vI8tuQ8FMI8Gi=hEeeu0xXdbba9frFj0=OqFfea0dXdd9vqai=hGuQ8kuc9pgc9s8qqaq=dirpe0xb9q8qiLsFr0=vr0=vr0dc8meaabaqaciGacaGaaeqabaqadeqadaaakeaacaWGsbWaa0baaSqaaiaadEgacaGGSaGaam4zaaqaaiaadogacaWGLbGaamOCaaaaaaa@3982@, Rg,gcan
 MathType@MTEF@5@5@+=feaafiart1ev1aaatCvAUfeBSjuyZL2yd9gzLbvyNv2Caerbhv2BYDwAHbqedmvETj2BSbqee0evGueE0jxyaibaiKI8=vI8tuQ8FMI8Gi=hEeeu0xXdbba9frFj0=OqFfea0dXdd9vqai=hGuQ8kuc9pgc9s8qqaq=dirpe0xb9q8qiLsFr0=vr0=vr0dc8meaabaqaciGacaGaaeqabaqadeqadaaakeaacaWGsbWaa0baaSqaaiaadEgacaGGSaGaam4zaaqaaiaadogacaWGHbGaamOBaaaaaaa@397A@. Each row in these matrices corresponds to the correlations between one gene and all other genes (with orthologs) from the same genome. Equivalent rows in the two matrices correspond to the correlation vectors of a pair of orthologs with all other orthologs from the respective genomes. The correlation between these vectors of correlations is defined as the initial estimation of expression conservation (*EC*_0_). EC scores are then iteratively refined by calculating weighted Pearson correlation coefficients (PCCw) where EC scores from the previous iteration are used as weights and genes with negative weights are excluded from the calculation. This procedure is repeated until convergence of the EC scores (*EC*_*k *_≈ *EC*_*k *- 1_). The iterative procedure can also be initiated from random weights to verify the convergence to a global minimum (see Materials and methods).

Ideally, we wish to compare the transcription responses of the *S. cerevisiae *genes to those of their *C. albicans *orthologs under the same set of conditions. However, the expression data of the two species was measured under different conditions and by different laboratories and could not be directly compared. We thus developed a novel method for comparing the expression profiles of two organisms, called 'iterative comparison of coexpression' (ICC; see Materials and methods and Figure 1). To analyze the expression conservation of an orthologous gene pair from *S. cerevisiae *and *Candida albicans *(*a*_*i*_^*cer *^and *a*_*i*_^*can*^, respectively), we compare their expression correlations with all other one-to-one orthologous pairs (*a*_*g*_^*cer*^, *a*_*g*_^*can*^; *g *= *1n*), as described below. This method follows the conceptual framework described by Ihmels *et al*. [16] and Dutilh *et al*. [17] and compares the architecture of the co-expression networks.

Dutilh *et al*. [17] defined expression conservation as the similarity between (i) the expression correlations between a gene from *S. cerevisiae *(*a*_*i*_^*cer*^) and all other *S. cerevisiae *genes (*a*_*g*_^*cer*^, *g *= 1..*n*), and (ii) the expression correlations between its ortholog from *Candida albicans *(*a*_*i*_^*can*^) and all other *Candida albicans *orthologs (*a*_*g*_^*can*^, *g *= 1..*n*), that is:

*EC*(*i*) = *PCC*(*R*_*i*,*g*_^cer^, *R*_*i*,*g*_^can^), *g *= *1*..*n*

where *PCC *is the Pearson correlation coefficient and *R*_*i*,*g*_^*cer *^is a vector of intra-species correlations, whose component *R*_*i*,*j*_^*cer *^is the correlation between the expression patterns of *a*_*i*_^*cer *^and *a*_*j*_^*cer *^(Figure 1). However, we note that a difference between *R*_*i*,*g*_^*cer *^and *R*_*i*,*g*_^*can *^does not necessarily correspond to a difference in the expression patterns of *a*_*i*_^*cer *^and *a*_*i*_^*can*^. For example, if *a*_*j*_^*cer *^and *a*_*j*_^*can *^have highly divergent expression profiles, then *R*_*i*,*j*_^*cer *^and *R*_*i*,*j*_^*can *^will be different even if the expression of *a*_*i*_^*cer *^and *a*_*i*_^*can *^has been completely conserved. Thus, when calculating the similarity between the vectors of correlations (*R*_*i*,*g*_^*cer *^and *R*_*i*,*g*_^*can*^), larger weight should be given to orthologous pairs whose expression has been conserved. In other words, when comparing a pair of orthologs, we would like to focus on their correlations with other orthologous pairs whose expression has been conserved.

To account for this effect, we employ an iterative algorithm that estimates expression conservation iteratively (see Figure 1 and Materials and methods). Briefly, at the first iteration, expression conservation is calculated as in Dutilh *et al*. [17]; at each subsequent iteration, the expression conservation values from the previous iteration are used as weights to calculate new expression conservation values. The iterative process proceeds until the expression conservation values converge.

### Expression conservation between *S. cerevisiae *and *C. albicans *orthologs

**Figure 2:**
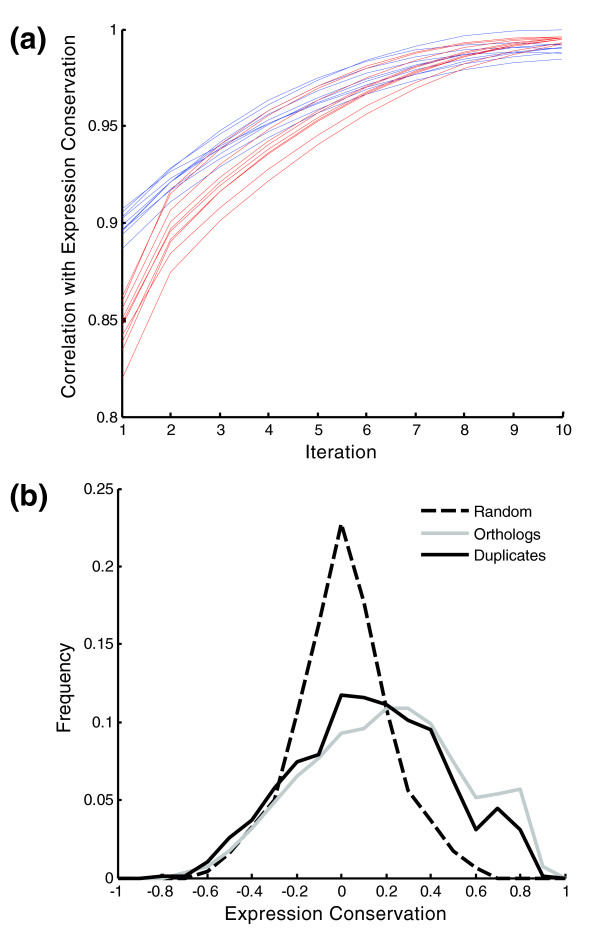
Expression conservation. **(a) **Controls for the ICC algorithm. ICC was applied ten times with randomly chosen initial weights (red), and ten times with randomly selected subsets of the expression conditions (blue). The correlations between these controls and expression conservation (from the original application) are shown after one to ten iterations. **(b) **Expression conservation values were calculated by ICC for all *S. cerevisiae*-*C. albicans *orthologs (solid black), duplicates from the WGD and their *C. albicans *ancestors (solid grey; both duplicates from each pair were compared to their ancestor), and randomly selected *S. cerevisiae*-*C. albicans *gene pairs (dashed black). Distributions are shown for bins of 0.1.

We applied ICC to the set of one-to-one orthologs between *S. cerevisiae *and *C. albicans *[18]. To this end, we assembled a large dataset of genome-wide expression data, consisting of approximately 1,700 expression profiles for *S. cerevisiae *and 244 expression profiles for *C. albicans *[16,19]. The results are summarized in Figure 2 and Additional data file 1.

Several tests were performed to validate the results. First, we ran the algorithm several times, starting from randomly chosen initial weights for each orthologous pair. In all cases the algorithm converged to the same results (Figure 2a), thus verifying the robustness of the iterative procedure. Second, we ran the algorithm with randomly chosen subsets of the expression datasets consisting of half the number of conditions for each species. Also in this case, the algorithm converged to the same results (Figure 2a). Third, we defined the set of conserved and divergent genes (5% highest or lowest expression conservation values, respectively) and examined their properties. Approximately 60% of the most conserved *S. cerevisiae *genes are essential [20] compared with 26% for all genes with orthologs in *C. albicans *(*p *< 10^-16 ^by the hypergeometric test). Furthermore, ribosome biogenesis was found to be the most enriched Gene Ontology (GO) term among the conserved genes (*p *< 10^-50 ^by hypergeometric test), whereas mitochondrion and mitochondrial ribosome were the most enriched GO terms among the divergent genes (*p *< 10^-17 ^for both by hypergeometric test). Indeed, these latter groups have undergone a large-scale adaptation of their expression profiles following the WGD [19]. Thus, the results of our algorithm are in good agreement with prior knowledge and expectations. Finally, we compared the distribution of expression conservation scores obtained for the orthologous pairs to that obtained for randomly chosen (non-orthologous) gene pairs. Expression conservation was higher for orthologs than non-orthologs (Figure 2b), indicating that the expression networks of the two yeast species have retained significant similarities.

### Comparison of duplicate gene pairs with their single orthologs

We next focused on the expression conservation of duplicate gene pairs. To this end, we used the expression conservation scores generated by the ICC for each of the one-to-one orthologs as weights to calculate the expression conservation of duplicate genes. Namely, for each duplicate gene pair in *S. cerevisiae*, we calculated two expression conservation scores between each of the duplicates and their single ortholog from *C. albicans *(see Materials and methods).

Out of the 457 duplicate pairs from the WGD event [2], we focused on 244 pairs compiled with the following two conditions. First, we performed a phylogenetic analysis and required that a single ortholog from *C. albicans *was predicted for both of the duplicates and that no other in-paralogs were found in *S. cerevisiae *(Materials and methods). This single ortholog serves as an out-group to estimate the expression of the ancestral gene before the WGD. Second, to avoid cases where the duplicates cross-hybridize to microarrays, thus leading to artificial correlations, we considered only duplicate pairs whose nucleotide sequence similarity was lower than 90%.

### Two modes of expression divergence for duplicates

**Figure 3:**
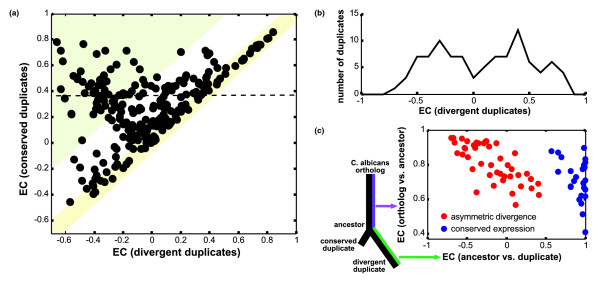
Expression conservation between duplicates and their ancestors. **(a) **Each dot represents the EC of a duplicate pair where the EC of the less conserved duplicate is shown on the x-axis and the EC of the more conserved duplicate is shown on the y-axis. The yellow region indicates similar rates of expression divergence (y - x < 0.2) and the green region indicates asymmetric rates of expression divergence (y - x > 0.5). The dashed line indicates a threshold of EC > 0.37, which is the 0.05 significance value from randomly selected *S. cerevisiae*-*C. albicans *gene pairs (see Figure 2b). **(b) **Distribution of EC for the least conserved duplicates among the 96 cases where the more conserved duplicate has significantly high EC (above the dashed line in (b)). **(c) **EC between the least conserved duplicate and the reconstructed ancestral expression (x-axis), and between the *C. albicans *ortholog and the reconstructed ancestral expression (y-axis). Red dots correspond to duplicates with asymmetric expression divergence (left peak in (b)) and blue dots correspond to duplicates with conserved expression (both copies with EC > 0.37).

As shown in Figure 3a, a large percentage of the duplicates appear to have evolved at a similar rate, as both gene pairs show similar expression conservation to their single *C. albicans *orthologs (for example, 79 duplicates in the yellow region). Notably, however, a similarly large fraction of duplicate pairs display distinctly different levels of expression conservation (for example, 63 duplicates in the green region). These cases indicate asymmetric rates of expression evolution among the two duplicate genes.

To further explore the distinction between duplicate pairs that evolve at similar versus asymmetric rates, we focused on the 96 duplicate pairs in which at least one of the copies has significantly high expression conservation (EC > 0.37; see dashed line in Figure 3a). This constraint removed cases for which it is difficult to infer the ancestral expression pattern, since the *C. albicans *expression pattern is much different from that of both duplicate genes. The expression conservation of the least conserved duplicates, in these cases, display a bimodal distribution with a boundary at approximately EC = 0 (Figure 3b). This distribution thus partitions the duplicate gene pairs into two classes.

The first class corresponds to duplicate gene pairs for which the expression of both copies resembles that of the *C. albicans *ortholog. Of these duplicate pairs, 28 have significantly high expression conservation for both copies; we refer to these as duplicate pairs with conserved expression. This class includes duplicate pairs whose divergence is probably related to other aspects of protein function, such as protein structure or interactions. In addition, duplicates in this class tend to have higher mRNA and protein abundance [21,22] than other duplicates (Additional data file 2), suggesting that some of these duplicate pairs could have been retained due to selection for high dosage.

Interestingly, in the second class, comprising 45% of the duplicate gene pairs (43 out of 96), one copy has a significant similarity (EC > 0.37) to the *C. albicans *ortholog (conserved), and the second copy has no similarity (EC < 0) to the *C. albicans *ortholog (divergent). The duplicate pairs displaying this asymmetric pattern of expression evolution are given in Table 1. This pattern is consistent with regulatory neofunctionalization, suggesting that the conserved copy has retained the ancestral function while the divergent copy performs a novel or altered function.

To verify the asymmetric divergence of these duplicate pairs we also performed an ancestral reconstruction analysis; since our method relies on correlations of expression with multiple genes, we performed a parsimony-based reconstruction [23] for each correlation value (see Materials and methods). This allowed us to decompose the expression divergence of each duplicate gene into two components: duplicate versus ancestor and ancestor versus *C. albicans *ortholog (Figure 3c). By definition, the ancestral reconstruction procedure tends to estimate an ancestral state that is an intermediate between the two duplicates. However, asymmetric expression divergence was still evident when examining the duplicate versus ancestor expression similarity (Table 1 and Figure 3c). In all cases, the expression similarity of the divergent copy was much lower than that of the conserved copy, and in most cases even lower than zero. Furthermore, the predicted ancestral expression patterns were more similar to the *C. albicans *patterns in duplicate pairs with asymmetric divergence compared to duplicate pairs with conserved expression (Figure 3c; *p *= 0.004 in a Wilcoxon rank sum test). This implies that expression of the duplicate pairs with asymmetric divergence is, in general, highly conserved, and divergence in these cases was restricted to one of the copies after duplication.

### Properties of duplicates predicted to undergo regulatory neofunctionalization

**Figure 4:**
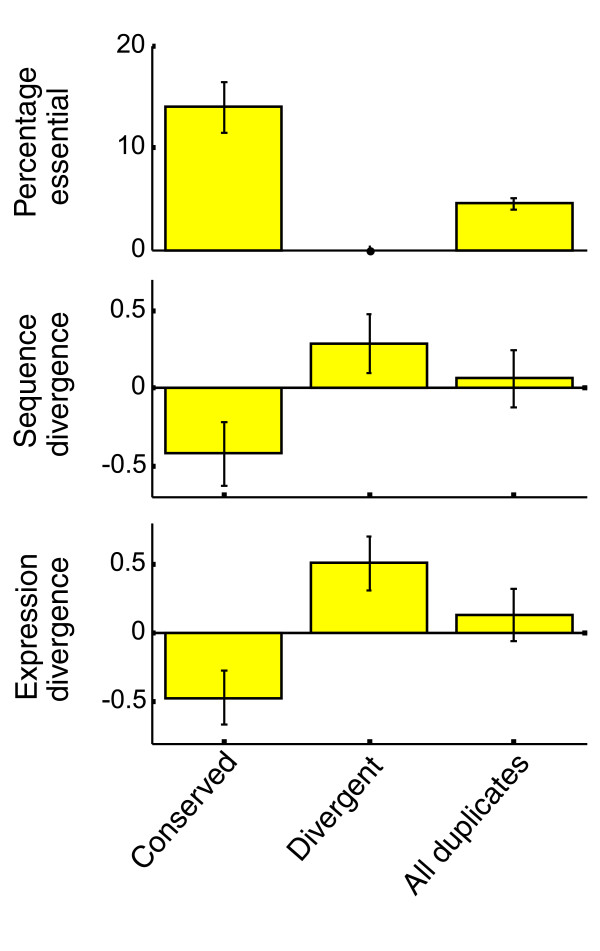
Properties of duplicates with predicted ancestral or novel functions. Duplicate gene pairs with asymmetric expression divergence are predicted to evolve by neofunctionalization, such that the copy with the higher EC (conserved) retained the ancestral function and the copy with the lower EC (divergent) performs a novel function. The percentage of essential genes, the average normalized sequence divergence [24] and the average normalized expression divergence [25] are shown for the conserved duplicates, divergent duplicates and all duplicates. Error-bars correspond to standard error, calculated by bootstrapping with 1,000 repeats.

Within a duplicate pair predicted to undergo regulatory neofunctionalization, our analysis distinguishes the conserved from the divergent copy. We next compared the set of conserved copies with that of the divergent copies using several datasets. First, we examined the fraction of essential genes [20] in the two gene sets. While eight of the conserved copies are essential, all of the divergent copies are dispensable (Figure 4). Second, we examined the extent of sequence variability [24], as well as expression variability [25], of these genes among the closely related *sensu-stricto *species, which diverged from *S. cerevisiae *long after the WGD. In both cases, the divergent copies were, on average, more variable than the conserved ones (Figure 4), indicating that they are still evolving rapidly. Taken together, these results suggest that the conserved copies typically perform stable and important functions, while the divergent copies are dispensable and undergoing continuous fine-tuning, as expected for newly derived functions.

### Whole-genome versus smaller-scale duplications

Recent studies have suggested that duplicate pairs arising from a WGD event have different characteristics to those arising from smaller-scale duplications [26-28]. To examine if this is the case with respect to gene expression evolution, we repeated the analysis presented above with 46 gene pairs from *S. cerevisiae *that were predicted to arise from small-scale duplications after speciation from *C. albicans *(see Materials and methods). Interestingly, only 1 duplicate pair had asymmetric expression divergence while 14 duplicate pairs had conserved expression (see Additional data file 3). This ratio is much different from the results in the WGD analysis where 43 duplicate pairs had asymmetric expression divergence and only 28 duplicate pairs had conserved expression. This difference may indicate that divergence of WGD duplicates is more likely to occur through regulatory divergence compared with small-scale duplicates.

### Divergence of protein sequence versus expression pattern

**Figure 5:**
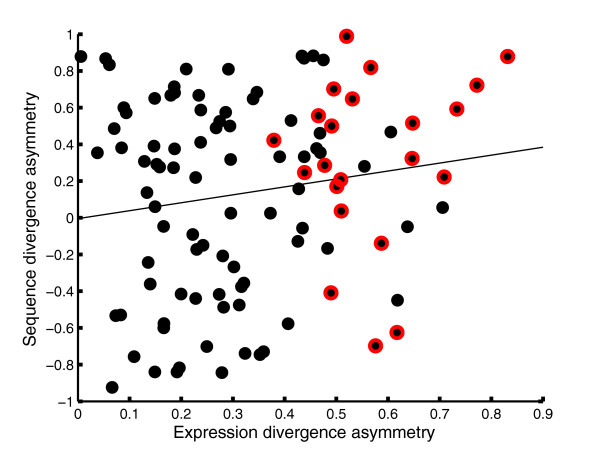
Asymmetric evolution of protein sequence and expression pattern. Scatter plot for asymmetry in expression and sequence divergence of the WGD duplicate pairs. Only duplicate pairs where more than 20 amino acid substitutions were predicted in at least one of the copies are included (see Materials and methods and Table 1). Red circles indicate duplicates with asymmetric expression divergence as defined in Figure 3. The line indicates the linear regression. The sign of sequence divergence asymmetry indicates the consistency between sequence and expression divergence (that is, negative values refer to duplicate pairs where the faster evolving copy in terms of sequence is the slower evolving copy in terms of expression).

We asked whether the observed asymmetry in the evolution of duplicates' expression patterns is correlated with asymmetric evolution of protein sequences [17,29,30]. To this end, we used a parsimony-based approach to asses the protein sequence divergence of each of the WGD duplicates from their pre-duplication ancestors (see Materials and methods and Table 1). We then compared the asymmetry of protein sequence divergence with that of expression divergence as estimated in the ancestral reconstruction analysis (Figure 5; see Materials and methods). The two measures of asymmetry are only weakly correlated (*r *= 0.15, *p *= 0.11). While most of the copies with asymmetric expression divergence also have high asymmetry of sequence divergence, others show similar levels of sequence divergence, and some even show an opposite trend where the divergent copy in terms of expression is more conserved in terms of sequence (negative sequence divergence asymmetry in Figure 5). These results suggest that although in many cases protein sequence and expression divergence are correlated, they represent distinct evolutionary mechanisms for the acquisition of novel functions.

## Discussion

We developed a new method for comparative analysis of genome-wide expression data (ICC) and applied it to characterize the diversification of yeast duplicates that originated at the WGD event. We identified a natural separation of duplicate pairs into two classes. The first class includes duplicates with symmetric expression divergence, such that both *S. cerevisiae *gene pairs displayed similar conservation with their *C. albicans *ortholog. The expression of many of these duplicate pairs is highly correlated (not shown), suggesting that they were retained by selection for high protein dosage or evolved through other functional aspects, such as protein structure or interactions.

The second class includes 43 duplicate gene pairs in which one copy showed a significant expression similarity to the *C. albicans *ortholog while the other copy displayed no similarity to the *C. albicans *ortholog. Some of these cases may represent neutral evolution of gene expression that has no functional significance. Alternatively, these cases may involve regulatory neofunctionalization. Although our method is not capable of detecting the action of directional selection, as required for neofunctionalization, the high conservation of one copy and the total lack of conservation of the other copy appear to be inconsistent with a neutral model. We thus interpreted this class as enriched with cases of regulatory neofunctionalization.

Another alternative interpretation is that this pattern indicates evolution by subfunctionalization, whereby the expression of the ancestral gene is partitioned between the two copies [4,11]. Our method does not compare the expression of duplicates and their orthologs under the same conditions, and thus subfunctionalization can lead to different patterns of expression conservation and is difficult to infer. In contrast, the neofunctionalization model clearly predicts that the gene with ancestral function will have high expression conservation, while the gene with derived function has low expression conservation. Our observations are, therefore, more consistent with the neofunctionalization model. It is important to note, however, that the neofunctionalization and subfunctionalization models are not mutually exclusive. For example, duplicates can evolve by subfunctionalization in terms of protein structure but by neofunctionalization in terms of expression profile. Furthermore, an initial subfunctionalization can be followed by neofunctionalization [31].

Our interpretation of neofunctionalization of the indicated genes is supported by their increased dispensability and enhanced variability in sequence and expression among closely related yeast species. Importantly, each of these 43 cases (Table 1) entails a prediction for the function of the ancestral protein in *C. albicans *and the evolutionary trajectory of the duplicate pair.

The new functions encoded by genes that evolved by neofunctionalization probably had an important role in the adaptation of yeast following the WGD. Perhaps the most significant adaptation of the *S. cerevisiae *lineage was the transformation from aerobic to predominantly anaerobic metabolism [32]. This adaptation involved the generation of novel pathways, most notably the repression of oxidative phosphorylation and related processes in the presence of glucose, known as glucose repression [33]. Indeed, of the duplicate pairs with asymmetric expression evolution at least two encode isoenzymes, and in these pairs the genes encoding the predicted novel function (HXK1 and PYK2) are under the control of the glucose repression pathway, while the genes encoding the predicted ancestral function (HXK2 and CDC19) are not repressed by glucose [34,35]. Another pair of isoenzymes (APA1 and APA2), which are ATP adenylyltransferases whose functional distinction is unclear, shows a similar pattern of regulation. The enzyme with the predicted novel function (APA2) is co-regulated with the anaerobic genes (expression correlation *r *= 0.4875 for HXK1 and *r *= 0.3966 for PYK2 in the *S. cerevisiae *dataset), while the enzyme with the predicted ancestral function (APA1) is co-regulated with the aerobic genes (expression correlation *r *= 0.5220 for HXK2 and *r *= 0.2940 for CDC19 in the *S. cerevisiae *dataset). This observation suggests that APA2 is the anaerobic ATP adenylyltransferase while APA1 is the aerobic one.

Neofunctionalization could also refine the function of existing complexes by creating specialized subunits with an elaborate regulation. For example, two of the duplicate gene pairs predicted to have evolved by neofunctionalization are alternative subunits of the same complex: EGD1 and BTT1 of the nascent polypeptide-associated complex [17,36], and FKS1 and GSC2 of beta-1,3-glucan synthase. Similarly, the transcription factors GZF3 (conserved) and DAL80 (divergent) are two regulators of nitrogen metabolism that can homo- or hetero-dimerize [37], presumably leading to different activities. These cases may provide examples where regulation of the alternative subunits' expression determines the composition of the complex at any cellular state, and thus dictates its condition-dependent function.

## Conclusion

Genes can evolve new functions by modulation of different characteristics, including the structure, physical interactions, expression patterns and localization of the proteins they encode. A comprehensive understanding of functional divergence thus requires an integrated analysis of different measures of divergence. Here, we studied the expression divergence of yeast duplicate pairs and identified 43 pairs with asymmetric divergence that is compatible with regulatory neofunctionalization. Importantly, most of these were not identified by sequence analysis [2] and, in general, the asymmetry of sequence divergence and that of expression divergence were only marginally correlated. Future studies will undertake the challenge of integrating these and other data types to provide a better understanding of the functional diversification of genes following duplications.

## Materials and methods

### Definition of homology relationships

The Inparanoid software [18] was used to identify one-to-one orthology between genes in *S. cerevisiae *and *C. albicans*. Duplicate pairs from the WGD were taken from Kellis *et al*. [2] and filtered with the following phylogenetic analysis: for each duplicate pair we constructed a clustalw multiple alignment of the duplicates, their single *K. waltii *ortholog (which was determined by synteny [2]) and all other matches from *S. cerevisiae *and *C. albicans *with a BLAST *p *value smaller than 10^-4^. These alignments were used to construct a neighbor-joining phylogenetic tree with the jukes-cantor distance, after ignoring gaps. We then demanded that each tree (or its subtree) contain only the pair of duplicates, the syntenic *K. waltii *ortholog and a single *C. albicans *ortholog. To further verify the *C. albicans *ortholog we also verified that the *K. waltii *ortholog and one of the duplicates are its best matches in the corresponding genomes.

The set of smaller-scale duplications was defined by: first, taking all duplications predicted by Inparanoid (that is, clusters of one *C. albicans *gene and two *S. cerevisiae *genes); second, excluding those that were predicted to arise from the WGD [2]; and third, filtering the remaining set using the phylogenetic analysis described above.

### Method for comparative analysis of gene expression

Expression datasets for *S. cerevisiae *and *C. albicans *containing multiple experimental conditions were collected as described in Ihmels *et al*. [16]. These expression matrices were restricted to genes for which orthology relationships were identified and ordered accordingly (that is, equivalent rows of the two matrices correspond to the expression profiles of a pair of orthologs). Next, these matrices were converted into correlation matrices by calculating, within each organism, the Pearson correlation coefficient (PCC) between the expression profiles of each pair of genes, over all the conditions. The resulting matrices (Rg,gcer
 MathType@MTEF@5@5@+=feaafiart1ev1aaatCvAUfeBSjuyZL2yd9gzLbvyNv2Caerbhv2BYDwAHbqedmvETj2BSbqee0evGueE0jxyaibaiKI8=vI8tuQ8FMI8Gi=hEeeu0xXdbba9frFj0=OqFfea0dXdd9vqai=hGuQ8kuc9pgc9s8qqaq=dirpe0xb9q8qiLsFr0=vr0=vr0dc8meaabaqaciGacaGaaeqabaqadeqadaaakeaacaWGsbWaa0baaSqaaiaadEgacaGGSaGaam4zaaqaaiaadogacaWGLbGaamOCaaaaaaa@3982@, Rg,gcan
 MathType@MTEF@5@5@+=feaafiart1ev1aaatCvAUfeBSjuyZL2yd9gzLbvyNv2Caerbhv2BYDwAHbqedmvETj2BSbqee0evGueE0jxyaibaiKI8=vI8tuQ8FMI8Gi=hEeeu0xXdbba9frFj0=OqFfea0dXdd9vqai=hGuQ8kuc9pgc9s8qqaq=dirpe0xb9q8qiLsFr0=vr0=vr0dc8meaabaqaciGacaGaaeqabaqadeqadaaakeaacaWGsbWaa0baaSqaaiaadEgacaGGSaGaam4zaaqaaiaadogacaWGHbGaamOBaaaaaaa@397A@) contain all the correlations between genes for which an orthology relationship was defined (*g *= 1..*n*). These matrices have similar dimensionality, and we proceeded by comparing equivalent rows:

EC0(i)=PCC(Ri,gcer,Ri,gcan)
 MathType@MTEF@5@5@+=feaafiart1ev1aaatCvAUfeBSjuyZL2yd9gzLbvyNv2Caerbhv2BYDwAHbqedmvETj2BSbqee0evGueE0jxyaibaiKI8=vI8tuQ8FMI8Gi=hEeeu0xXdbba9frFj0=OqFfea0dXdd9vqai=hGuQ8kuc9pgc9s8qqaq=dirpe0xb9q8qiLsFr0=vr0=vr0dc8meaabaqaciGacaGaaeqabaqadeqadaaakeaacaWGfbGaam4qamaaBaaaleaacaaIWaaabeaakiaacIcacaWGPbGaaiykaiabg2da9iaadcfacaWGdbGaam4qaiaacIcacaWGsbWaa0baaSqaaiaadMgacaGGSaGaam4zaaqaaiaadogacaWGLbGaamOCaaaakiaacYcacaWGsbWaa0baaSqaaiaadMgacaGGSaGaam4zaaqaaiaadogacaWGHbGaamOBaaaakiaacMcaaaa@4A24@

This corresponds to the initial estimation of expression conservation (EC) in which identical weights are given to the correlations with all genes. We then iteratively refined this measure by calculating a weighted correlation, where the weight for a correlation with each gene is given by the EC of that gene from the previous iteration:

ECk(i)=PCCw(Ri,g′cer,Ri,g′can)
 MathType@MTEF@5@5@+=feaafiart1ev1aaatCvAUfeBSjuyZL2yd9gzLbvyNv2Caerbhv2BYDwAHbqedmvETj2BSbqee0evGueE0jxyaibaiKI8=vI8tuQ8FMI8Gi=hEeeu0xXdbba9frFj0=OqFfea0dXdd9vqai=hGuQ8kuc9pgc9s8qqaq=dirpe0xb9q8qiLsFr0=vr0=vr0dc8meaabaqaciGacaGaaeqabaqadeqadaaakeaacaWGfbGaam4qamaaBaaaleaacaWGRbaabeaakiaacIcacaWGPbGaaiykaiabg2da9iaadcfacaWGdbGaam4qaiaadEhacaGGOaGaamOuamaaDaaaleaacaWGPbGaaiilaiqadEgagaqbaaqaaiaadogacaWGLbGaamOCaaaakiaacYcacaWGsbWaa0baaSqaaiaadMgacaGGSaGabm4zayaafaaabaGaam4yaiaadggacaWGUbaaaOGaaiykaaaa@4B6E@

where:

PCCw(X,Y)=∑wi(Xi−X¯)(Yi−Y¯)∑wi(Xi−X¯)2∑wi(Yi−Y¯)2
 MathType@MTEF@5@5@+=feaafiart1ev1aaatCvAUfeBSjuyZL2yd9gzLbvyNv2Caerbhv2BYDwAHbqedmvETj2BSbqee0evGueE0jxyaibaiKI8=vI8tuQ8FMI8Gi=hEeeu0xXdbba9frFj0=OqFfea0dXdd9vqai=hGuQ8kuc9pgc9s8qqaq=dirpe0xb9q8qiLsFr0=vr0=vr0dc8meaabaqaciGacaGaaeqabaqadeqadaaakeaacaWGqbGaam4qaiaadoeacaWG3bGaaiikaiaadIfacaGGSaGaamywaiaacMcacqGH9aqpdaWcaaqaamaaqaeabaGaam4DamaaBaaaleaacaWGPbaabeaakiaacIcacaWGybWaaSbaaSqaaiaadMgaaeqaaOGaeyOeI0Yaa0aaaeaacaWGybaaaiaacMcacaGGOaGaamywamaaBaaaleaacaWGPbaabeaakiabgkHiTmaanaaabaGaamywaaaacaGGPaaaleqabeqdcqGHris5aaGcbaWaaOaaaeaadaaeabqaaiaadEhadaWgaaWcbaGaamyAaaqabaGccaGGOaGaamiwamaaBaaaleaacaWGPbaabeaakiabgkHiTmaanaaabaGaamiwaaaacaGGPaWaaWbaaSqabeaacaaIYaaaaaqabeqaniabggHiLdGcdaaeabqaaiaadEhadaWgaaWcbaGaamyAaaqabaGccaGGOaGaamywamaaBaaaleaacaWGPbaabeaakiabgkHiTmaanaaabaGaamywaaaacaGGPaWaaWbaaSqabeaacaaIYaaaaaqabeqaniabggHiLdaaleqaaaaaaaa@5EC7@

*w*_*i *_= *EC*_*k *- 1_(*i*)

*g*' = {*l *∈ *g *| *EC*_*k *- 1_(*l*) > 0}

This procedure was repeated until convergence:

∑i∈g[ECk(i)−ECk−1(i)]2<0.1
 MathType@MTEF@5@5@+=feaafiart1ev1aaatCvAUfeBSjuyZL2yd9gzLbvyNv2Caerbhv2BYDwAHbqedmvETj2BSbqee0evGueE0jxyaibaiKI8=vI8tuQ8FMI8Gi=hEeeu0xXdbba9frFj0=OqFfea0dXdd9vqai=hGuQ8kuc9pgc9s8qqaq=dirpe0xb9q8qiLsFr0=vr0=vr0dc8meaabaqaciGacaGaaeqabaqadeqadaaakeaadaaeqbqaaiaacUfacaWGfbGaam4qamaaBaaaleaacaWGRbaabeaakiaacIcacaWGPbGaaiykaiabgkHiTiaadweacaWGdbWaaSbaaSqaaiaadUgacqGHsislcaaIXaaabeaakiaacIcacaWGPbGaaiykaiaac2faaSqaaiaadMgacqGHiiIZcaWGNbaabeqdcqGHris5aOWaaWbaaSqabeaacaaIYaaaaOGaeyipaWJaaGimaiaac6cacaaIXaaaaa@4B29@

Finally, to validate the iterative heuristic, we calculated EC scores when the initial weights were randomly selected:

EC0(i)=PCCw(Ri,gcer,Ri,gcan)
 MathType@MTEF@5@5@+=feaafiart1ev1aaatCvAUfeBSjuyZL2yd9gzLbvyNv2Caerbhv2BYDwAHbqedmvETj2BSbqee0evGueE0jxyaibaiKI8=vI8tuQ8FMI8Gi=hEeeu0xXdbba9frFj0=OqFfea0dXdd9vqai=hGuQ8kuc9pgc9s8qqaq=dirpe0xb9q8qiLsFr0=vr0=vr0dc8meaabaqaciGacaGaaeqabaqadeqadaaakeaacaWGfbGaam4qamaaBaaaleaacaaIWaaabeaakiaacIcacaWGPbGaaiykaiabg2da9iaadcfacaWGdbGaam4qaiaadEhacaGGOaGaamOuamaaDaaaleaacaWGPbGaaiilaiaadEgaaeaacaWGJbGaamyzaiaadkhaaaGccaGGSaGaamOuamaaDaaaleaacaWGPbGaaiilaiaadEgaaeaacaWGJbGaamyyaiaad6gaaaGccaGGPaaaaa@4B20@

where *w*_*i *_= *rand*([0,1]).

This was repeated ten times; in each case the algorithm described above was applied until convergence and the EC scores were compared to those without randomization. In all cases the results from the randomized procedure were similar to those of the original procedure (*PCC *> 0.99), indicating that the original results reflect a global minimum.

### Application to duplicate gene pairs

After EC scores were computed for all ortholog gene pairs, these scores were used as weights for a similar analysis of duplicates. For each pair of duplicates from *S. cerevisiae *and their orthologs from *C. albicans*, we calculated two EC scores for comparison of each of the duplicates with their ortholog.

### mRNA and protein abundance

mRNA abundance averaged over various studies was taken from Beyer *et al*. [22], and protein abundance was taken from Ghaemmaghami *et al*. [21]. These values were log_2_-tranformed, and then centered and normalized.

### Ancestral reconstruction of expression correlations

Each gene in *S. cerevisiae *and *C. albicans *is represented in our analysis by its expression correlation with a reference set of one-to-one orthologs. Thus, for each pair of duplicates and each reference gene, we performed ancestral reconstruction to infer the correlation of the ancestral gene (before duplication) with the reference gene. Ancestral reconstruction is done with a parsimony-based procedure [23], which uses the correlation values in each of the duplicates and the *C. albicans *ortholog to infer the ancestral correlation that minimizes the total divergence of that value. The inferred correlations with the entire reference set defines the ancestral expression pattern that is then compared to the duplicate pair and the *C. albicans *ortholog using the EC score defined above.

### Variability of protein sequence and expression profiles

Variability of protein sequences (adjusted Ka/Ks) among four yeast species from the *Saccharomyces sensu-stricto *complex were taken from [17], transformed as in the original study (*f*(*k*) = Log [*k *+ 0.001]), and normalized by subtracting their mean and dividing by their standard deviation. Variability of expression profiles in response to environmental stresses among four yeast species from the *Saccharomyces sensu-stricto *complex were taken from [18].

### Protein sequence divergence

Multiple alignments of the duplicates and their single orthologs from *K. waltii *and *C. albicans *were used to estimate protein sequence divergence using a parsimony-based approach. Namely, each position with the same amino acid in the *K. waltii *ortholog, the *C. albicans *ortholog and at least one of the duplicates was assumed to represent the ancestral state before duplication; if the second duplicate had a different amino acid at that position, then a substitution was inferred. The number of substitutions inferred for each duplicate gene is used as an estimate of protein sequence divergence (Table 1).

### Asymmetry of sequence and expression divergence

Asymmetry was defined as x1−x2x1+x2
 MathType@MTEF@5@5@+=feaafiart1ev1aaatCvAUfeBSjuyZL2yd9gzLbvyNv2Caerbhv2BYDwAHbqedmvETj2BSbqee0evGueE0jxyaibaiKI8=vI8tuQ8FMI8Gi=hEeeu0xXdbba9frFj0=OqFfea0dXdd9vqai=hGuQ8kuc9pgc9s8qqaq=dirpe0xb9q8qiLsFr0=vr0=vr0dc8meaabaqaciGacaGaaeqabaqadeqadaaakeaadaWcaaqaaiaadIhadaWgaaWcbaGaaGymaaqabaGccqGHsislcaWG4bWaaSbaaSqaaiaaikdaaeqaaaGcbaGaamiEamaaBaaaleaacaaIXaaabeaakiabgUcaRiaadIhadaWgaaWcbaGaaGOmaaqabaaaaaaa@3CBC@, where x_1 _and x_2 _are measures of divergence of the duplicate gene pair. For sequence divergence x_i _represented the number of amino acid substitutions and for expression divergence it was 1 - EC. For each gene pair, x_1 _was chosen as the copy with lower expression conservation, such that asymmetry of expression divergence is always positive and the sign of asymmetry of sequence divergence reflects the congruence between sequence and expression analyses (negative asymmetry of sequence divergence means that the copy with lower expression conservation had higher sequence conservation). Note that this measure is not equivalent to that used to detect extreme cases of asymmetry where we demanded that one copy has EC > 0.372 and the other copy has EC < 0.

## Additional data files

The following additional data are available with the online version of this paper. Additional data file 1 is a table that lists the expression conservation values of 2,644 orthologous pairs from *S. cerevisiae *and *C. albicans*. Additional data file 2 is a figure showing the high mRNA and protein abundance of duplicated genes with conserved expression compared with other duplicated genes. Additional data file 3 is a figure showing the expression conservation of duplicated genes from small-scale duplication events. In contrast to duplicates from the WGD, there is only one case of asymmetric divergence and many cases of conserved expression.

## Supplementary Material

Additional data file 1 Expression conservation values of 2,644 orthologous pairs from *S. cerevisiae *and *C. albicans*. Click here for file

Additional data file 2 High mRNA and protein abundance of duplicated genes with conserved expression compared with other duplicated genes. Click here for file

Additional data file 3 In contrast to duplicates from the WGD, there is only one case of asymmetric divergence and many cases of conserved expression. Click here for file

## Figures and Tables

**Table 1:** Duplicate pairs with asymmetric expression evolution

Conserved*	Divergent*	Ortholog*	EC_1_^†^	EC_2_^†^	AA_1_^‡^	AA_2_^‡^
URA7	URA8	orf19.3941	0.83877	-0.70319	19	12
YML125C	YML087C	orf19.7307	0.78458	-0.67655	31	5
YGR141W	YPR157W	orf19.1800	0.71756	-0.65682	11	15
URA5	URA10	orf19.2555	0.65222	-0.62355	14	2
SEC14	YKL091C	orf19.941	0.56525	-0.57108	30	3
YDR018C	YBR042C	orf19.137	0.63182	-0.55743	6	26
UTH1	NCA3	orf19.5032	0.6716	-0.53582	25	8
MRS3	MRS4	orf19.2178	0.59171	-0.5275	3	3
YDR341C	MSR1	orf19.3341	0.86239	-0.49736	31	2
SVL3	PAM1	orf19.1139	0.51523	-0.48769	32	21
YGL060W	YBR216C	orf19.5034	0.53514	-0.43043	28	26
YKL035W	YHL012W	orf19.1738	0.55403	-0.41198	178	1
FKS1	GSC2	orf19.2929	0.53428	-0.40078	24	17
APA1	APA2	orf19.5630	0.71784	-0.38482	7	9
YOR054C	SIS2	orf19.7378	0.59512	-0.38274	13	12
EGD1	BTT1	orf19.1154	0.80319	-0.35655	18	3
PRK1	YNL020C	orf19.2605	0.56058	-0.3195	14	14
YOR227W	YPL137C	orf19.6544	0.66576	-0.28599	31	41
GSP1	GSP2	orf19.5493	0.70457	-0.26817	1	0
YBL054W	YER088C	orf19.2545	0.78265	-0.25468	9	19
EMP70	YDR107C	orf19.2746	0.44981	-0.22268	32	13
YDR185C	MSF1'	orf19.3089	0.72298	-0.21596	3	19
GZF3	DAL80	orf19.2842	0.59906	-0.20028	3	2
YEL006W	YIL006W	orf19.1393	0.6866	-0.16477	5	28
ARE2	ARE1	orf19.2248	0.82595	-0.13257	39	10
YMR102C	YKL121W	orf19.7235	0.59001	-0.12411	21	6
YIL036W	YER045C	orf19.6102	0.60972	-0.08006	8	2
YJL084C	YKR021W	orf19.5605	0.57884	-0.0794	43	26
YOR108W	LEU4	orf19.6086	0.80251	-0.04464	6	16
YKR027W	CHS6	orf19.5155	0.54734	-0.03258	9	17
YHR149C	YGR221C	orf19.1426	0.65	0.010312	27	15
YGL133W	YPL216W	orf19.5510	0.67582	0.050333	33	11
YER119C	YBL089W	orf19.1210	0.84012	0.06238	22	14
RSC6	SNF12	orf19.2265	0.68101	0.068833	13	31
HXK2	HXK1	orf19.542	0.71034	0.10264	18	9
YLL010C	YLR019W	orf19.5406	0.82661	0.11215	13	8
AAP1'	APE2	orf19.5197	0.59717	0.14552	7	11
YGL144C	YDL109C	orf19.3991	0.82365	0.17816	41	21
PMT2	PMT3	orf19.6812	0.75012	0.26011	57	10
NHP6A	YBR089CA	orf19.4623.3	0.78273	0.3078	1	0
MKK2	MKK1	orf19.6889	0.77227	0.31153	7	10
YBR238C	YGL107C	orf19.7459	0.69723	0.40045	2	0
CDC19	PYK2	orf19.3575	0.81763	0.4044	56	12

*'Conserved' and 'Divergent' refer to the duplicates from *S. cerevisiae *with high and low expression similarity to the ortholog from *C. albicans*, respectively. ^†^Expression conservation between the conserved (EC_1_) and divergent (EC_2_) copies and their reconstructed ancestor. ^‡^Number of amino acid substitutions between the conserved (AA_1_) and divergent (AA_2_) copies and their reconstructed ancestor.
