# Supplementary material for: Comparative analysis indicates regulatory neofunctionalization of yeast duplicates
Source: Genome Biol. 2007 Apr 5;8(4):R50. doi: 10.1186/gb-2007-8-4-r50 (PMC1895995; doi:10.1186/gb-2007-8-4-r50)
Supplement: Additional data file 2 — High mRNA and protein abundance of duplicated genes with conserved expression compared with other duplicated genes. [file gb-2007-8-4-r50-S2.pdf]

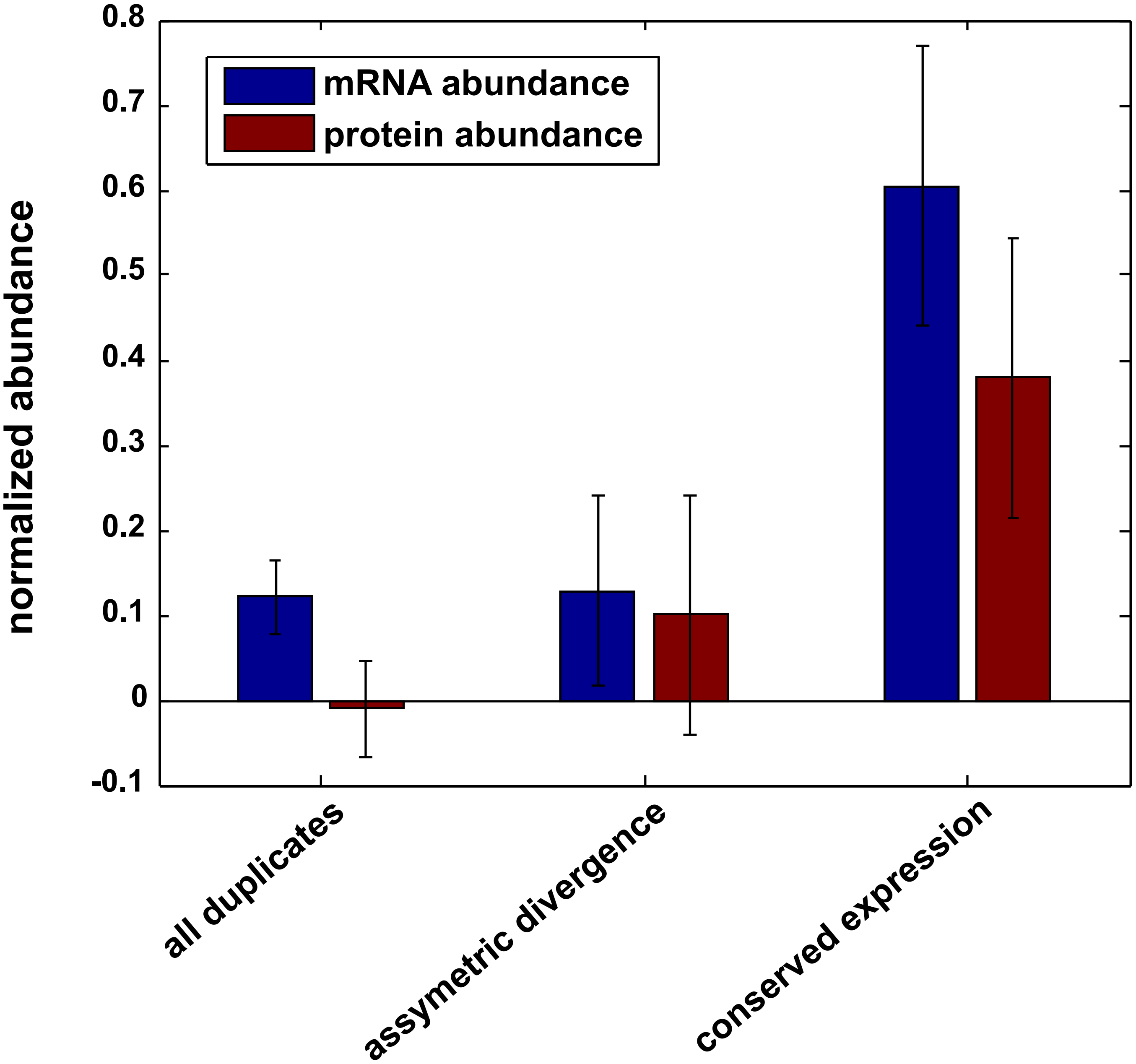

Additional data file 2. mRNA and protein abundance of duplicate genes. mRNA and protein abundance were log2-transformed and then normalized to have mean zero and unit Std.The average normalized mRNA and protein abundance are shown for all duplicates, the 43 duplicate pairs with asymmetric divergence and the 28 duplicate pairs with conserved expression. Error-bars were calculated by bootstrapping with 1000 repeats. The average abundance of duplicate pairs with conserved expression is significantly higher than that of all duplicates ($p<0.05$ for both mRNA and protein abundance in t-test).
