# Supplementary material for: Comparative analysis indicates regulatory neofunctionalization of yeast duplicates
Source: Genome Biol. 2007 Apr 5;8(4):R50. doi: 10.1186/gb-2007-8-4-r50 (PMC1895995; doi:10.1186/gb-2007-8-4-r50)
Supplement: Additional data file 3 — In contrast to duplicates from the WGD, there is only one case of asymmetric divergence and many cases of conserved expression. [file gb-2007-8-4-r50-S3.pdf]

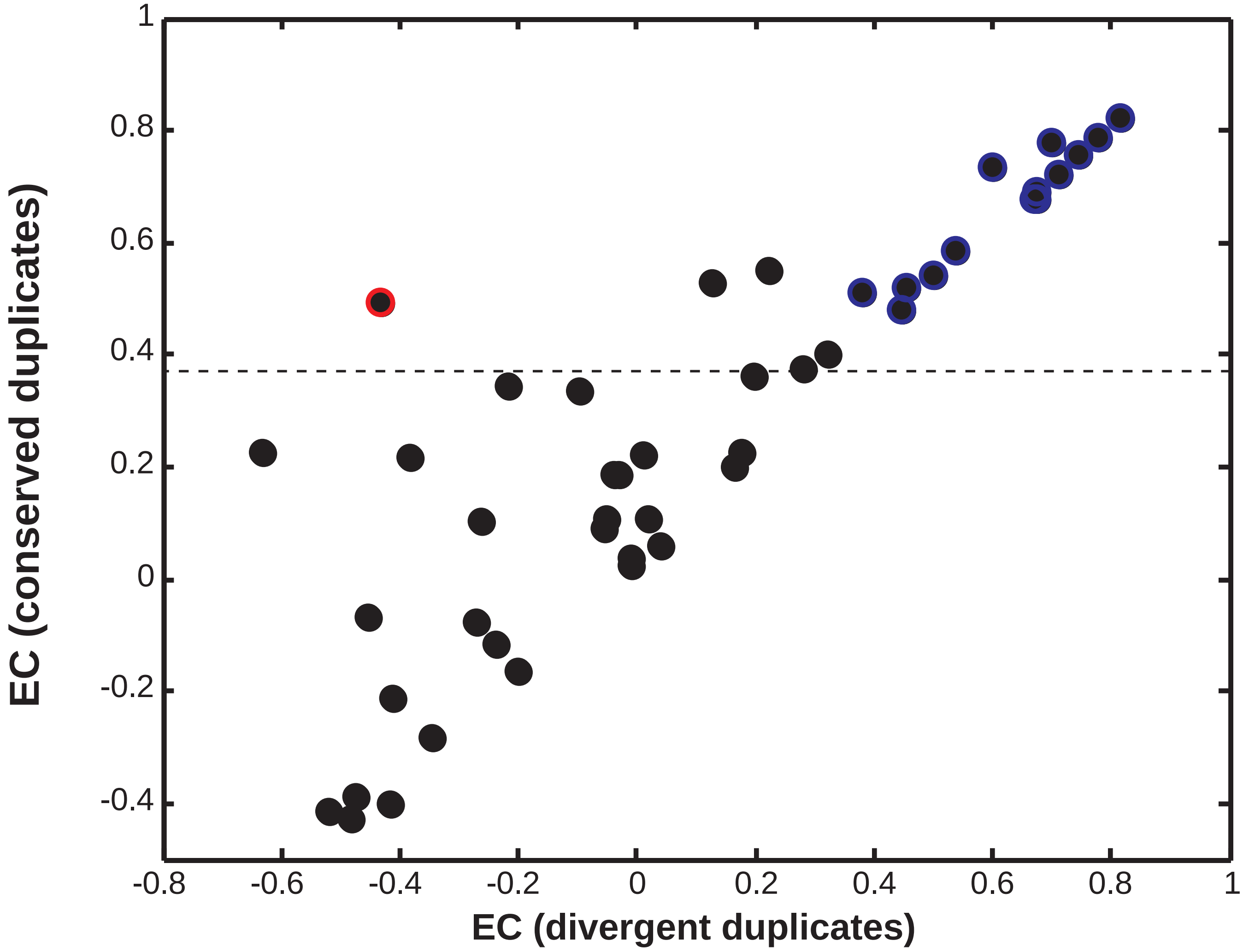

Additional data file 3. Expression Conservation between duplicate pairs from small-scale duplication events and their single C.albicans orthologs. Expression conservation was calculated for each of the 46 small-scale duplication pairs with their C. albicans single orthologs.Expression conservation of the more conserved copies are shown at the *y*-axis and those of the more divergent copies are shown at the *x*-axis. Dashed line represent the 0.05 significance threshold of EC>0.37; blue and red circles represent duplicate pairs with conserved expression and asymmetric divergence, respectively.
